# Impact of climate change on the spread of fascioliasis into the extreme south of South America

**DOI:** 10.1371/journal.pntd.0013433

**Published:** 2025-08-18

**Authors:** Pablo Fernando Cuervo, Roberto Mera y Sierra, Patricio Artigas, María Cecilia Fantozzi, María Dolores Bargues, Santiago Mas-Coma

**Affiliations:** 1 Departamento de Parasitología, Facultad de Farmacia, Universidad de Valencia, Valencia, Spain; 2 CIBER de Enfermedades Infecciosas, Instituto de Salud Carlos IIII, Madrid, Spain; 3 Centro de Investigación en Parasitología Regional (CIPAR), Universidad Juan Agustín Maza, Mendoza, Argentina; James Cook University Faculty of Science and Engineering: James Cook University College of Science and Engineering, AUSTRALIA

## Abstract

The impact of global warming on the transmission of fascioliasis, a highly pathogenic zoonotic snail-borne disease, was already highlighted during the 2010’s. However, since then, only a few studies have tried to relate the climatic change with the uprise of outbreaks in endemic areas of animal or human fascioliasis. This might be because assessing the consequences of a changing climate on the spread of fascioliasis is extremely challenging, as it presents the widest latitudinal, longitudinal and altitudinal distribution known for a snail-borne disease. In the Americas, where it is only caused by *Fasciola hepatica*, the disease is widespread throughout the continent, except in its southernmost extremity in the Patagonia region, which was believed to be due to the too low temperatures. Though, recent empirical evidence indicates an ongoing spread of the disease into more southern latitudes. The present study aims to assess the long-term evolution of climate change factors and forecast indices throughout this extreme South American region to conclude whether their impact might have been the cause of the southward expansion of the fascioliasis endemic area. The use of seasonal-trend decomposition analyses and of spatial interpolation techniques demonstrated a remarkable climatic change in the Patagonia region allowing to clarify the southern spread of the disease. This is the first study highlighting a clear link between the consequences of a changing climate and the spread of a fascioliasis endemic area and its transmission risk to extreme latitudes. Moreover, it provides some crucial recommendations and concerns regarding the application and interpretation of two widely applied climatic forecast indices. If current climate trends persist, this geographical expansion is expected to progress further. These findings not only provide critical insight into local disease dynamics but also underscore the broader implications of climate-driven changes in the distribution of snail-borne diseases globally.

## Author summary

Fascioliasis is a highly pathogenic parasitic disease caused by liver flukes and transmitted by freshwater snails from animals to humans. Despite the presaged global warming’s impact on its transmission, only a few studies have tried to verify it. This might be because assessing this impact is extremely challenging, as this disease presents a worldwide distribution. In the Americas, the disease is widespread throughout the continent, except in its southernmost extremity in the Patagonia region, which was believed to be due to the too low temperatures. Though, recent reports indicate that the disease is spreading into more southern latitudes. We analyzed the long-term evolution of climate factors to assess if their impact might have been the cause of the southward expansion of fascioliasis in this extreme South American region. Our detailed analyses demonstrated a remarkable climatic change in the Patagonia region and allowed to clarify the southern spread of the disease. This is the first study linking the consequences of a changing climate with the spread of fascioliasis to extreme latitudes, warning about how the distribution and transmission of snail-borne diseases may be modified due to influences of climate change in other world regions in the immediate future.

## 1. Introduction

Fascioliasis is a highly pathogenic disease caused by *Fasciola hepatica* in every continent excepting Antarctica, while in regions of Africa and Asia it is also caused by *F. gigantica* [1]. Both trematodes are transmitted by freshwater snails mainly of the family Lymnaeidae [2]. Its significant economic and sanitary impact is well-known in veterinary medicine due to the high losses it causes in husbandry worldwide [3]. However, it is far from being under control [4,5]. Moreover, this zoonotic disease constitutes a serious problem of public health, with an estimated 17 million people infected throughout the five continents [6]. The increasing importance of human fascioliasis relies on its high pathogenicity [7,8] and immunosuppressive capacity [9,10]. Indeed, fascioliasis is listed among the Foodborne Trematodiases prioritized by the World Health Organization (WHO) in the Neglected Tropical Diseases Roadmaps for 2020 and 2030 [11,12].

Fascioliasis’ current state of global emergence/re-emergence [6,13] appears to be related to the impact of climate and global change [14–17]. The association of fascioliasis with a changing environment relies on the high dependence of both fasciolid larval stages and their freshwater lymnaeid snail vectors on climatic and environmental characteristics [17,18]. Therefore, fascioliasis has been identified as one of the diseases to be affected in more complex ways by climate and global changes [19].

In South America, as throughout the continent, fascioliasis is caused only by *F. hepatica* [1], whereas the absence of *F. gigantica* has been linked to the absence of its specific lymnaeid vector species of the *Radix* group [20]. In its turn, *F. hepatica*, together with its main lymnaeid snail vector *Galba truncatula*, was introduced from southwestern Europe into South America by the Spanish initial colonizers when transporting livestock along the trans-Atlantic trips [13]. A deep multidisciplinary analysis did already rule out the suggested previous existence of *F. hepatica* in the present Argentinian Patagonia based on liver fluke egg findings in 2300-year-old fossil coprolites from autochthonous sylvatic cervids [1,21]. The colonization of South America initially took place by entering with livestock through the western coast of the Pacific Ocean around 500 years ago. Originally from the high Andean altitudes, cattle [22], donkeys [23] and mules [24] were the animals most used for the transport of the mineral (i.e., silver and gold) down to the Atlantic port of Buenos Aires for its further trans-ocean transport to Spain [25]. Such long intra-continental caravan migrations accelerated the diffusion into more southern latitudes of Argentina and Chile of both *F. hepatica* [1,24,25] and *G. truncatula* [26,27]. In these two countries, an authochthonous lymnaeid snail species of the same *Galba*/*Fossaria* group, such as *Lymnaea viator*, further facilitated the fasciolid establishment and additional southward spread into colder zones as those of the Patagonia region [6].

Human endemic areas of fascioliasis have been described in Andean regions, mainly in high altitude areas of countries such as Bolivia, Peru, Chile, Ecuador and Venezuela [1]. Despite the numerous studies showing similarities in physiography, climate and lymnaeid species composition with endemic areas in Bolivia, Perú and Chile, human fascioliasis in Argentina is evidently in need for additional studies [28]. A retrospective analysis highlights that its real epidemiological situation in high risk rural, mainly altitudinal areas, may currently be underestimated [28]. In fact, the description in the last years of some human hyperendemic areas revealed a far more complex situation than previously thought [29,30].

Concerning animal fascioliasis in Argentina, it is ranked as the fourth disease of veterinary importance [31] and has been reported throughout the country, except in the southernmost colder extreme of the continent [28,32]. Besides the evident economic losses, the emergence of triclabendazole resistance, both in cattle and sheep [31,33], represents a major concern as it is the antihelminthic drug of choice for the treatment of liver fluke infection of humans [21].

Widespread in most humid areas of north and central Patagonia, fascioliasis was absent from the extreme of southern Patagonia, in the provinces of Santa Cruz and Tierra del Fuego. In the 1990’s, its southern distribution was reported to be limited near latitude 46° S [34]. Yet, about a decade later, transmission was verified up to latitude 48° S [32]. Moreover, after several years, the presence of livestock infected with *F. hepatica* was demonstrated about 200 km to the south, reaching the latitude 50° S [35]. This empirical evidence indicates an ongoing spread of the disease into more southern latitudes, which might be attributed to the dispersal of fasciolids and lymnaeids with livestock movements [1,13,25]. The question is why this southward spread is occurring in the present instead of throughout the long previous period of 500 years. So far, the absence of the liver fluke in the southernmost extremity of the continent was believed to be due to the too low temperatures preventing fascioliasis transmission to adapt to such cold extreme latitudes.

It appears now to be logical to think at a potential impact of global warming as the underlying cause allowing for the establishment of long-term transmission foci of fascioliasis in southernmost areas. The present study aims to analyze the evolution of local climate change factors of the last 65-year period throughout this extreme South American region to conclude whether their impact might have been the cause of the southward expansion of the fascioliasis endemic area into most austral Patagonia.

## 2. Methods

### 2.1. Study area

**Fig 1:**
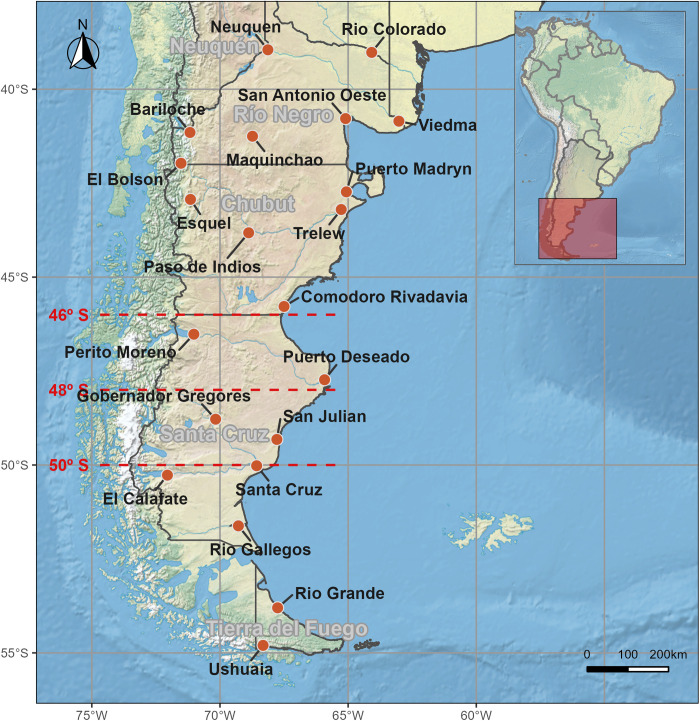
Study area, indicating the location of the meteorological stations analyzed, and the area where the southern expansion of *F. hepatica* occurred during the last decades, between latitudes 46° and 50° S (red dotted lines). Base layer image by Natural Earth is in the public domain (https://www.naturalearthdata.com/about/terms-of-use/), and countries geopolitical shapes from GADM are freely available for academic use and other non-commercial use (https://gadm.org/license.html).

The study was focused on the Patagonia region, located in southern Argentina (38-54.9° S, 62.5-73.6° W) and delimited to the north by the Colorado River (Fig 1). The Argentinean Patagonia region is mainly characterized as a semi-desert, with average annual rainfall between 100 and 500 mm, and annual mean temperature that varies from 13.4 °C in the north to 5 °C in the south [36,37]. Mean annual precipitation is mainly concentrated in winter and most of the precipitation events resulted in less than 5 mm [37]. Its spatial variability is mostly explained by the distance from the Andes [38]. A particular emphasis was placed on the area where the southern expansion of *F. hepatica* seems to have occurred during the last decades, between parallels 46° and 50° S (Fig 1).

Northern and central Argentinean Patagonia are characterized by temperate climates with moderate to high rainfall, as well as the presence of wetlands, streams, and flood-irrigated meadows, conditions favorable for sustaining lymnaeid snail populations, the intermediate hosts of *F. hepatica* [34,35,39]. Livestock production in these areas is predominantly extensive, based on year-round grazing of natural pastures [40]. Sheep are the principal livestock species, with an estimated population of 5.9 million, followed by goats and cattle, the latter numbering approximately 950,000 [41]. These environmental conditions, combined with high livestock densities and extensive grazing practices, increase the environmental contamination with *Fasciola* eggs and promote disease transmission. In contrast, the southernmost provinces, such as Santa Cruz and Tierra del Fuego, exhibit colder and drier climates that are less suitable for sustaining the parasite’s life cycle [34,35].

### 2.2. Climatic data

Daily climatic data, including temperature (maximum [t_max_] and minimum [t_min_]) and precipitation (prcp), was requested to the *Servicio Meteorológico Nacional* from Argentina (www.smn.gov.ar). The information received included data from every meteorological station (n = 21) actively monitored by the *Servicio Meteorológico Nacional* in the study region for, in most cases, a 65-year period (1956–2021) (Fig 1 and Table 1).

**Table 1:** Meteorological stations analyzed in the study area, and respective daily data available (analyzed period and coverage %).

Station	Province	Geographical coordinates	Altitude (m)	Available data (period [%])
Neuquén	Neuquén	38.95° S–68.13° W	271	1956–2021 [96.2]
Río Colorado	Río Negro	39.02° S–64.08° W	79	1956–2019 [86.6]
San Antonio Oeste	Río Negro	40.78° S–65.10° W	20	1988–2019 [91.9]
Viedma	Río Negro	40.85° S–63.02° W	7	1967–2021 [95.7]
Puerto Madryn	Río Negro	42.73° S–65.07° W	136	1992–2019 [87.7]
Maquinchao	Río Negro	41.25° S–68.73° W	888	1956–2021 [96.8]
Bariloche	Río Negro	41.15° S–71.17° W	840	1956–2021 [97.2]
El Bolsón	Río Negro	41.97° S–71.52° W	337	1978–2019 [81.5]
Esquel	Chubut	42.93° S–71.15° W	797	1961–2021 [94.0]
Trelew	Chubut	43.20° S–65.27° W	43	1956–2021 [95.8]
Paso de Indios	Chubut	43.82° S–68.88° W	460	1968–2019 [91.4]
Comodoro Rivadavia	Chubut	45.78° S–67.50° W	46	1956–2021 [97.6]
Perito Moreno	Santa Cruz	46.52° S–71.02° W	429	1956–2017 [71.9]
Puerto Deseado	Santa Cruz	47.73° S–65.92° W	80	1956–2019 [92.2]
Gobernador Gregores	Santa Cruz	48.78° S–70.17° W	358	1956–2019 [82.1]
San Julián	Santa Cruz	49.32° S–67.78° W	62	1956–2021 [89.8]
Santa Cruz	Santa Cruz	50.02° S–68.57° W	111	1958–2019 [53.9]
El Calafate	Santa Cruz	50.27° S–72.05° W	204	2000–2019 [96.9]
Río Gallegos	Santa Cruz	51.62° S–69.28° W	19	1956–2021 [95.0]
Río Grande	Tierra del Fuego	53.80° S–67.75° W	22	1959–2021 [87.4]
Ushuaia	Tierra del Fuego	54.80° S–68.32° W	57	1990–2021 [92.5]

**Fig 2:**
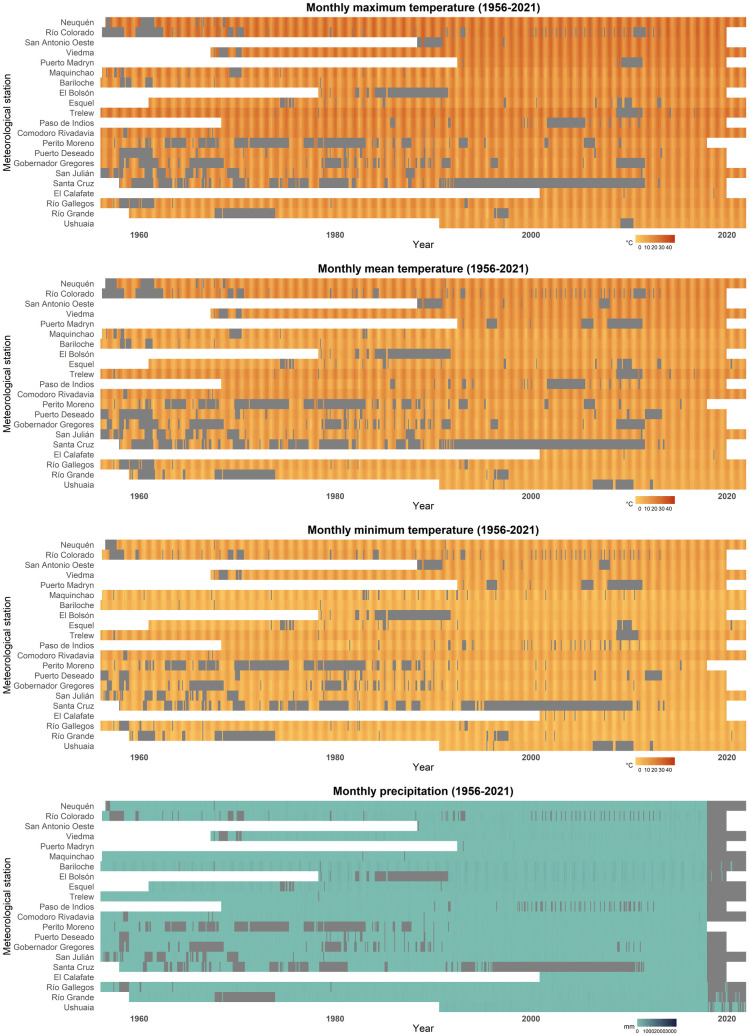
Heatmaps indicating the monthly data availability of the main climatic factors (maximum temperature, mean temperature, minimum temperature, and precipitation) relevant for the calculation of the fascioliasis forecast indices for the 1956–2021 period for each meteorological station. The colour ramps indicate the magnitude of the climatic factor, and the grey colour indicates the absence of data. For an improved readability, it is suggested to visualize this figure in its digital version.

**Fig 3:**
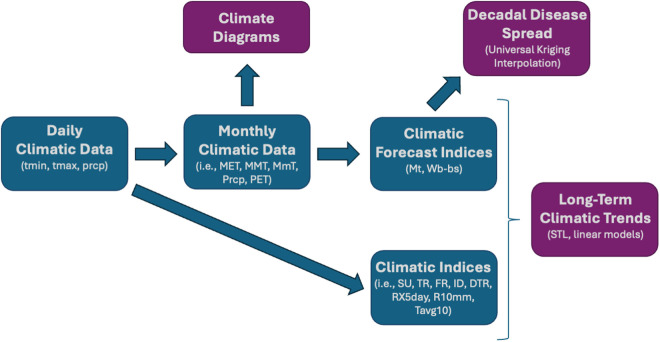
Flowchart illustrating the main methodological and data processing steps. Main outputs are colored in purple.

Monthly climatic data was calculated from the daily data aforementioned, including: mean environmental temperature (MET), mean maximum temperature (MMT), mean minimum temperature (MmT), extreme maximum temperature (EMT), extreme minimum temperature (EmT), maximum temperature difference (MTD; i.e., MMT - MmT), and extreme temperature difference (ETD; i.e., EMT - EmT), all in °C; total precipitation (Prcp) in mm, number of days with precipitation (DP; i.e., prcp >=1), number of days with frost (DF; i.e., t_min_ < 0 °C), and total potential evapotranspiration (PET) in mm [42]. Monthly climatic data from each meteorological station studied is summarized in S1 Table and its availability is presented in Fig 2. The main methodological and data processing steps are illustrated in Fig 3.

Typical climate and seasonality at each meteorological station was assessed by representing mean monthly data of the aforementioned factors in climate diagrams (Fig 3). Climate diagrams illustrate the year-round profile of monthly average values for temperature and precipitation, providing a brief summary of average climatic conditions. Climate diagrams, based on proposed models [43], were produced for each meteorological station considered using a modified script from the R package *climatol* (https://climatol.eu/). Roughly, when the precipitation curve undercuts the temperature curve, the area in between them indicates dry season, whereas when the precipitation curve supersedes the temperature, the curve indicates moist season.

### 2.3. Climatic indices

In order to provide an accurate and reliable analysis of changes in climate extremes in the study area, a suite of indices defined by the Expert Team on Climate Change Detection (ETCCDI, http://etccdi.pacificclimate.org/indices.shtml, Table 2) and based on daily data were calculated (Fig 3): total precipitation (Prcptot), mean of t_min_ (MT_min_, often corresponding to night-time temperature) and t_max_ (MT_max_, often corresponding to daytime temperature), count of frost days (FD), count of summer days (SU), count of icing days (ID), count of tropical nights (TR), count of dry (CDD) and wet days (CWD), and daily temperature range (DTR). In addition, two indices describing precipitation frequency (R10mm and R20mm) and one describing intensity (RX5day) were analyzed (Table 2). Further, the count of days surpassing a mean temperature of 10 °C (Tavg10) was calculated as a measure of the number of days allowing for fascioliasis transmission (see below in Climatic Forecast Indices).

**Table 2:** Definition of climate change indices.

Name	Definition	Unit	Resolution
*Precipitation indices*			
Prcptot	total precipitation in wet days (mm)	mm	annual
RX5day	maximum consecutive 5-day precipitation (mm)	mm	monthly
R10mm	number of days when prcp = 10mm	count	annual
R20mm	number of days when prcp = 20mm	count	annual
CDD	length of dry spell (maximum number of consecutive days with prcp < 1mm)	count	annual
CWD	length of wet spell (maximum number of consecutive days with prcp = 1mm)	count	annual
*Temperature indices*			
MT_max_	mean maximum temperature	°C	annual
MT_min_	mean minimum temperature	°C	annual
DTR	daily temperature range (mean difference between t_max_ and t_min_)	°C	monthly
FD	frost days, number of days when t_min_ < 0 °C	count	annual
SU	summer days, number of days when t_max_ > 25 °C	count	annual
ID	icing days, number of days when t_max_ < 0 °C	count	annual
TR	tropical nights, number of days when t_min_ > 20 °C	count	annual
Tavg10	transmission days, number of days when t_mean_ >=10 °C	count	annual

These selected climate indices offer valuable insights for assessing fascioliasis risk under varying climatic scenarios, as they reflect environmental factors influencing both the development of *F. hepatica* and the ecology of its intermediate lymnaeid snail hosts [17,18]. Total precipitation (Prcptot) and the count of wet days (CWD) are associated with sustained moisture levels in soils and surface water bodies, promoting snail habitat stability and parasite development, while a high number of consecutive dry days (CDD) may desiccate these habitats, reducing transmission potential. The mean of minimum (MT_min_) and maximum temperatures (MT_max_), along with the daily temperature range (DTR), inform on thermal suitability, with moderate and stable temperatures supporting parasite and snail survival, whereas extreme variability may hinder life cycle completion. The frequency of frost (FD) and icing days (ID) is particularly relevant in southern latitudes, where cold conditions can suppress or eliminate snail populations. In contrast, the count of summer days (SU) and tropical nights (TR) may indicate thermal stress in warmer areas, potentially limiting snail activity. Finally, precipitation frequency indices (R10mm and R20mm) and the intensity index RX5day help characterize rainfall patterns that can either enhance transmission by sustaining wet environments or, in the case of extreme rainfall events, redistribute parasite eggs across the landscape.

### 2.4. Climatic forecast indices

Since the incidence of fascioliasis infection in the definitive host has been related to air temperature, rainfall, and/or potential evapotranspiration [17], several climatic forecast indices have been proposed to estimate the risk of transmission. The two most useful indices have proved to be the Wet Day index or Mt index [44] and later improved [45,46], and the Water budget-based system index or Wb-bs index [47] and later modified for large-scale regional use [48].

The Wet Day index (Mt) [44] is expressed by the equation:


$$Mt=\frac{n(R-PET+125)}{25}$$

where *n* is the number of rainy days, *R* is the rainfall in mm, and *PET* is the potential evapotranspiration in mm [45,46]. This index is calculated considering only those months in which the MET is ≥ 10 °C, as it is the minimum temperature required for the development of *F. hepatica* [44,49]. Mt values sufficient to support transmission have been considered as ≥ 100 in UK, 80 in France [45,46], and as low as 55–60 in Pakistan [14].

The Water-budget-based system (Wb-bs), adapted for regional use with monthly climate data, was calculated following Malone et al. (1998) [48]:


$$Wb-bs=(GDD*days\;in\;month),\;if\;[R-(PET*0.8)]>0,+(GDD*z)*\frac{R-PET}{25},\;if\;(R-PET)>0$$

where *R* is the rainfall, *PET* the potential evapotranspiration, *z* the number of surplus rainy days in the month (calculated as the mode for each particular station), and *GDD* the growing degree-days calculated as the monthly MET minus the base development temperature for *F. hepatica*, which is 10 °C [44,49]. In the first part of the formula, subtracting the factor PET*0.8 from rainfall *R* is equivalent to counting monthly GDD if rain-dependent moisture storage is present in the top 2.5 cm layer of a soil water budget model [48]. The second part counts GDD if monthly surplus water is present due to rainfall events [48]. The Wb-bs index is calculated based on accumulative values in a continuous way when different from 0. Conventionally established risk values are: 600 = no risk; 601–1500 = low risk; 1500–3000 = moderate risk; and 3000 = high risk [14,42,50,51].

For both climatic forecast indices, potential evapotranspiration (PET) was calculated according to the method proposed by Hargreaves and Samani (1985) [52] and modified by Samani (2000) [53]. This method requires minimal climatic data (temperature and solar radiation), and its results are closely correlated with those obtained by the frequently proposed Penman method [54,55]. Further, it has already been used for the estimation of PET in Patagonia [56].

**Modifications to the Wb-bs index for semi-desert and desert environments:** The calculation of the Wb-bs index was adjusted to explicitly account for the distinct soil moisture dynamics of the Patagonian semi-desert, where rainfall is generally insufficient to generate transient water bodies due to a consistently negative rainfall-to-PET ratio [37]. By excluding the original rainfall-dependent moisture storage condition if [R−(PET*0.8)]>0, the modified index reflects the ecological fact that fascioliasis transmission in this region does not depend on ephemeral pools, but rather on hydrologically stable moist meadows sustained by shallow groundwater and topographic runoff, locally known as *mallines* [34,57]. These modifications allow the index to more accurately estimate GDD accumulation under conditions where surface moisture persists independent of rainfall events, thus maintaining suitable microhabitats for the development of both *F. hepatica* and its intermediate snail hosts despite the arid macroclimate.

Summarizing, the modified Wb-bs index was calculated as:


$$Wb-bs=(GDD*days\;in\;month)+[(GDD*z)*\frac{R-PET}{25},\;if\;(R-PET)>0]$$

Climatic forecast indices were calculated for each month of each year (Fig 3), producing a data set from which the following was obtained for each station: (i) monthly values for the 1956–2021 period; (ii) monthly means for the 1956–2021 period; (iii) annual means and mean annual maximum values for each year from 1956 to 2021, and (iv) mean maximum value for each decade in the period analyzed (i.e., 1961–1970, 1971–1980, 1981–1990, 1991–2000, 2001–2010, 2011–2020).

### 2.5. Analysis of the long-term variation of climatic factors, climatic indices and forecast indices

The influence of the climate change is difficult to detect within time series that are heavily influenced by seasonal climatic variations [58]. Thus, a Seasonal-Trend decomposition procedure (STL) based on locally weighed regression (Loess) [59] was applied to reveal potentially significant trends in climatic factors, climatic indices and forecast indices [16] (Fig 3). The STL procedure decomposes the time series into trend, seasonal and remainder components. Then, linear models were used to analyze the trend component of each variable (Fig 3). The trend component was considered as the response, whereas “time” was included as the explanatory variable to account for the long-term variation in time-series data. The STL procedure and linear models were used with the entire dataset (annually) and with the four seasons: winter (JJA, June-July-August), spring (SON, September-October-November), summer (DJF, December-January-February), and autumn (MAM, March-April-May). Results were considered statistically significant when *p-value* <0.05. To depict the significant trends detected in the area and compare between locations, the magnitude of change was expressed per decade.

### 2.6. Analysis of the southern geographical spread of the disease

The southern progression of the disease was analyzed by obtaining the geographical transmission threshold for each decade in the period 1956–2021 by means of spatial interpolation techniques [7] (Fig 3). Based on the Wb-bs mean maximum values calculated for each decade and each meteorological station, continuous layers covering the Patagonia region were obtained for each decade between 1961 and 2020 (a total of six layers). Spatial interpolation was performed by means of universal kriging. Universal kriging was selected over other alternatives because this technique explicitly accounts for the spatial autocorrelation using a variogram. Additionally, it allows to include one or more independent variables as covariates to further model the spatial variation in the data. In this case, a digital elevation model was used as covariate for altitude correction. Subsequently, the contour corresponding to the transmission threshold (Wb-bs index value = 600) was extracted from each continuous layer to visualize the southern spread of the endemic area.

### 2.7. Spatial and statistical analyses

All the necessary calculations, spatial analyses, interpolation techniques and statistics have been carried out with R Statistical Software (‘R: A language and environment for statistical computing’, version 4.2.2 [2022-10-31 ucrt], http://www.r-project.org) and RStudio 2022.02.3.492 (‘RStudio: Integrated development environment for R’, http://www.rstudio.com/). Results were considered statistically significant when *p-value* <0.05.

## 3. Results

### 3.1. Seasonality of climatic factors and climatic forecast indices

**Fig 4:**
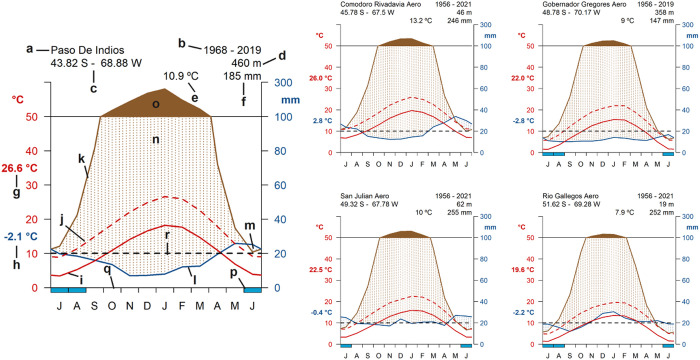
Climate diagrams during the 65-year period analyzed (1956-2021) of five meteorological stations, representing the latitudinal and longitudinal geographical gradient of the area where the geographical spread of the disease has been recently described. Each plot indicates: (a) the station; (b) the period of years represented by the data; (c) the geographical coordinates in decimal degrees; (d) altitude in meters; (e) the mean yearly temperature; (f) the mean yearly precipitation; (g) the mean maximum temperature during the warmest month; (h) the mean minimum temperature during the coldest month; (i) the mean monthly temperature curve; (j) the mean maximum monthly temperature curve; (k) the potential evapotranspiration as proposed by Hargreaves and Samani (1985) [52]; (l) the mean monthly precipitation curve; (m) the wet and (n) dry seasons; (o) the months when the mean monthly potential evapotranspiration exceeds 100 mm; (p) the months with a mean minimum temperature under 0 °C; (q) the mean duration of the period without freezing and (r) the 10 °C temperature threshold below which fascioliasis transmission due to *F. hepatica* is unlikely. Months labels in the X-axis are presented as July (J), August (A), September (S), October (O), November (N), December (D), January (J), February (F), March (M), April (A), May (M) and June (J).

The main climatic factors influencing fascioliasis prevalence and transmission (temperature and precipitation) were analyzed by means of climate diagrams and mean annual data (Fig 4 and S1 Table). The climate diagrams (Fig 4) reveal distinct seasonal and regional patterns in temperature and precipitation that are highly relevant to fascioliasis transmission. Overall, the region exhibits a marked aridity, as indicated by a consistently negative rainfall/PET balance across all sites (Fig 4). This imbalance signifies that PET consistently exceeds precipitation, resulting in limited surface water retention and the near absence of rain-fed temporary aquatic habitats. Consequently, the transmission of *F. hepatica* and the survival of its lymnaeid snail hosts is largely restricted to permanent moist habitats supported by groundwater discharge or snowmelt. Vegetation cover is generally sparse and dominated by xerophytic species, further reducing soil moisture retention and contributing to the fragmentation of suitable habitats. Annual mean temperatures hover around 9 °C, with MET barely reaching 15 °C during the summer months (December to February), a period that also coincides with the dry season (Fig 4 and S1 Table). This seasonal overlap suggests limited moisture availability during the warmer months when both the parasite and snail hosts are most metabolically active, thus constraining the potential transmission window. Conversely, the short humid season from May to July is characterized by low temperatures (MET < 5 °C; minimum temperatures often below 0 °C) (Fig 4 and S1 Table), which are suboptimal for both snail activity and parasite development. These climatic dynamics highlight the ecological dependence of fascioliasis transmission on hydrologically stable microenvironments and the tight coupling of risk periods to narrow seasonal and thermal windows.

**Fig 5:**
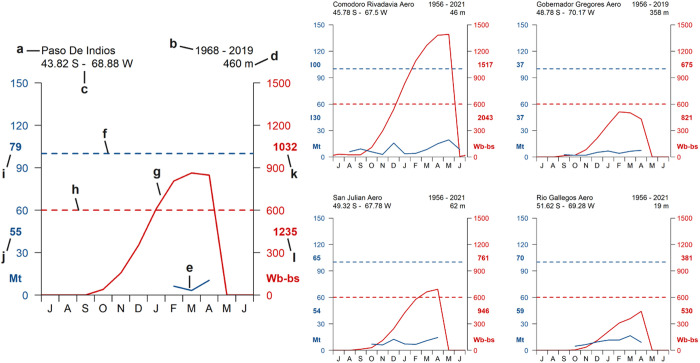
Monthly evolution during the 65-year period analyzed (1956-2021) of the climatic forecast indices of five meteorological stations included in the study, representing the latitudinal and longitudinal geographical gradient of the area where the geographical spread of the disease has been described recently. Each plot indicates: (a) the station; (b) the period of years represented by the data; (c) the geographical coordinates in decimal degrees; (d) altitude in meters; (e) the mean monthly curve of the Wet-day indicator (Mt index); (f) the critical transmission threshold of the Mt index; (g) the mean monthly curve of the cumulative Water-Budget-Based System indicator (Wb-bs index); (h) the critical transmission threshold of the Wb-bs index; (i) the mean yearly value of the Mt index; (j) the maximum monthly value recorded of the Mt index; (k) the mean yearly value of the cumulative Wb-bs index; (l) and the maximum monthly value recorded of the cumulative Wb-bs index. Months labels in the X-axis are presented as July (J), August (A), September (S), October (O), November (N), December (D), January (J), February (F), March (M), April (A), May (M) and June (J).

The seasonality of the climatic forecast indices is strongly determined by temperature, which is below 10 °C during most of the year. Thus, fascioliasis transmission can occur during summer and autumn, but more pronouncedly in the latter. In general, the monthly values reached by the Mt index are barely sufficient to sustain fascioliasis transmission in the region (Fig 5). Concerning the Wb-bs index, the transmission threshold of 600 is easily reached northward from the parallel 46° S. In the area where the geographical spread of the disease has been recently described, *a priori* transmission seems not to be possible. Yet, when the Wb-bs values were analyzed in detail (monthly maximum values), values reaching the level to support transmission were observed in several periods (see the long-term analysis below). In Gobernador Gregores the monthly maximum value recorded reached a value of 821, while in San Julian the monthly value recorded reached the value of 946 (Fig 5). In both cases, peaks were observed between March and April.

### 3.2. Analysis of the long-term variation of climatic factors, climatic indices and forecast indices

**Fig 6:**
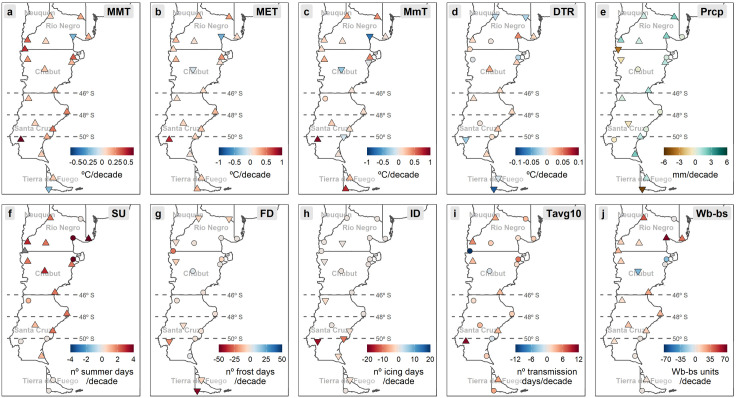
Spatial distribution in the Patagonia region of annual trends during the 65-year period analyzed (1956-2021) for (a) mean maximum temperature (MMT), (b) mean environmental temperature (MET), (c) mean minimum temperature (MmT), (d) precipitation (Prcp), (e) daily temperature range (DTR), (f) summer days (SU), (g) frost days (FD), (h) icing days (ID), (i) transmission days (Tavg10) (j) and Wb-bs index. The gradient of colors denotes the intensity of change. Stations with trends significant at the 0.05 level are marked with an upward or downward triangle denoting positive and negative trends, respectively. Circles depict non-significant trends. Country’s geopolitical shapes from GADM are freely available for academic use and other non-commercial use (https://gadm.org/license.html). For an improved readability, it is suggested to visualize this figure in its digital version.

After removing the yearly cycle with the STL procedure, a significant major climatic change was demonstrated in the entire southern endemic area (Fig 6). Annual trends in maximum, mean and minimum temperatures show significant increases in most of the stations (Fig 6a, 6b and 6c), whereas the daily temperature range (DTR) has increased in most locations (Fig 6d). Annual trends in precipitation are positive in most of the endemic region (Fig 6e), particularly in the coastal area. The region has experienced a significant increase in the count of summer days (SU) (Fig 6f), while the frequency of frost and icing days (FD, ID) has been reduced (Fig 6g, 6h). The number of days allowing for fascioliasis transmission (Tavg10) has increased significantly (Fig 6i), and thus the Wb-bs climatic forecast index has also increased in most cases, except for the central plateau (Fig 6j).

**Fig 7:**
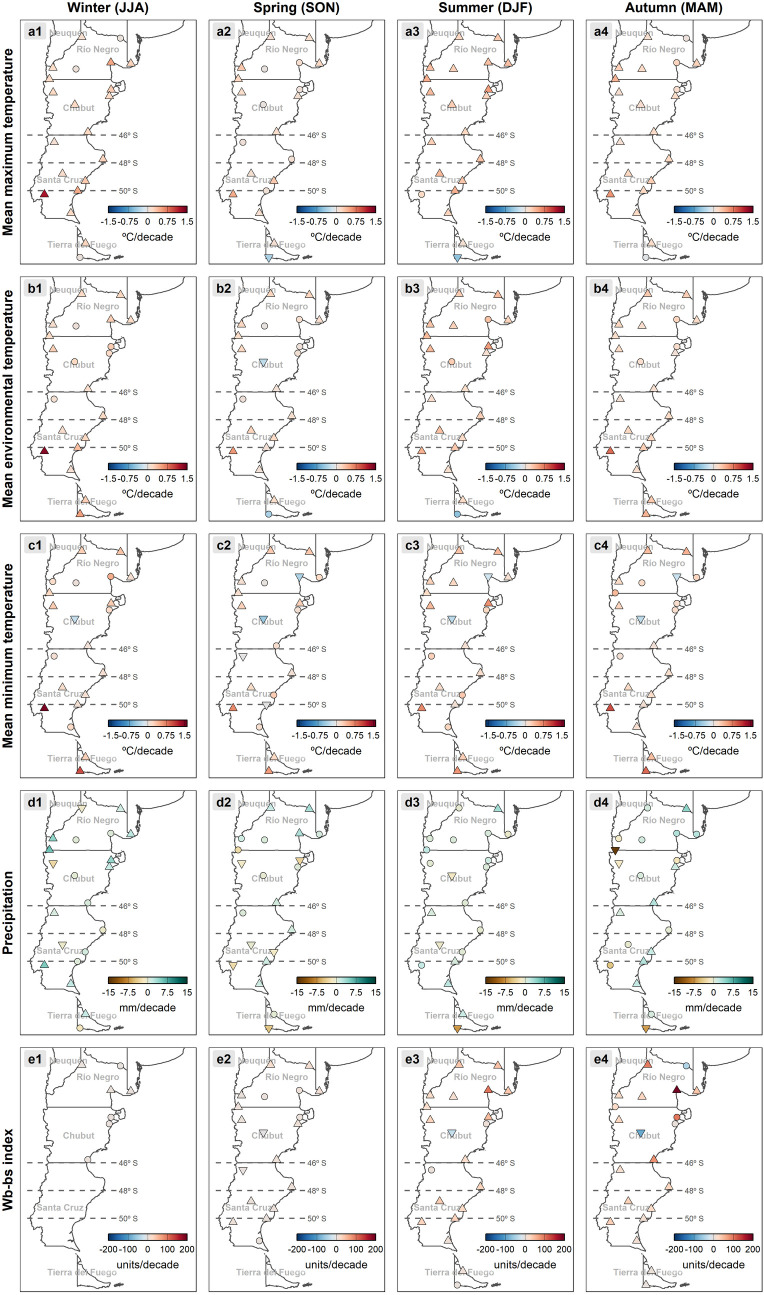
Spatial distribution in the Patagonia region of seasonal trends during the 65-year period analyzed (1956-2021) for (a) mean maximum temperature, (b) mean environmental temperature, (c) mean minimum temperature, (d) precipitation, and (e) Wb-bs index. The gradient of colors denotes the intensity of change. Stations with trends significant at the 0.05 level are marked with an upward or downward triangle denoting positive and negative trends, respectively. Circles depict non-significant trends. Country’s geopolitical shapes from GADM are freely available for academic use and other non-commercial use (https://gadm.org/license.html). For an improved readability, it is suggested to visualize this figure in its digital version.

Temperature seasonal trends significantly increased throughout all seasons in most of the locations analyzed (Fig 7a, 7b and 7c), being more evident in maximum and mean temperatures. Seasonal trends in precipitation are highly variable and not significant in many of the localities (Fig 7d). Besides winter, when no transmission is possible, the climatic risk indices have increased significantly in the rest of seasons (Fig 7e), being especially evident during autumn.

**Fig 8:**
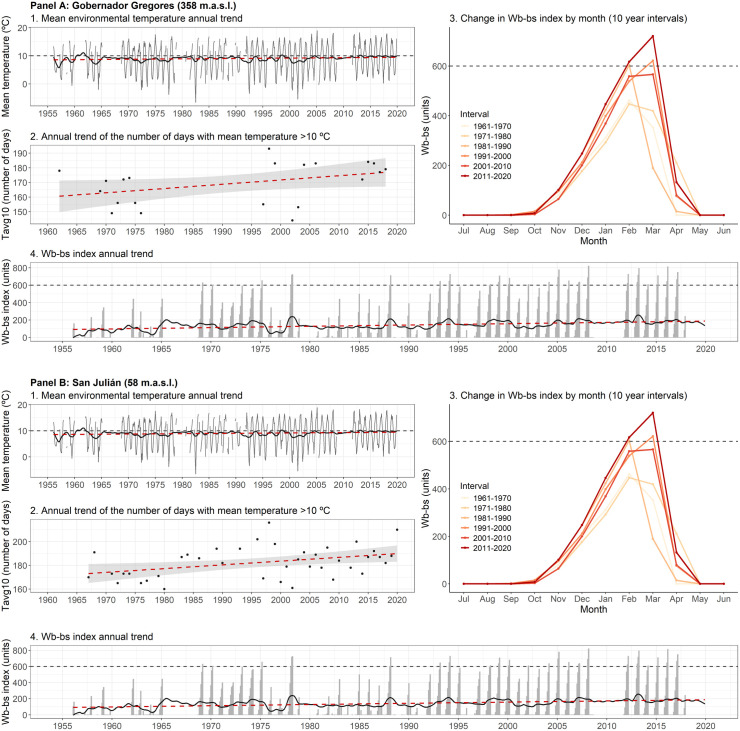
Annual change during the 65-year period analyzed (1956-2021) of mean environmental temperature and Wb-bs index in two selected meteorological locations (Gobernador Gregores and San Julian) placed between latitudes 48° and 50° S, where the geographical spread of the fascioliasis transmission area has occurred. In each panel, picture **(1)** shows the mean environmental temperature annual trend, represented by the observed values (grey columns), the observed trend obtained from the Seasonal-Trend decomposition procedure (black line), and the regression line (red-slashed line) (the slashed grey line depicts the 10 °C threshold required for the life cycle of *Fasciola hepatica* to progress). Picture **(2)** shows the trend of the annual number of days with mean temperature above 10 °C, represented by the observed values (black dots), and the regression line obtained from generalized linear models (red-slashed line). Picture **(3)** represents the mean monthly change of the Wb-bs index by intervals of 10 years (the slashed grey line depicts the value of 600, which indicates risk of transmission. Picture **(4)** shows the Wb-bs index annual trend, represented by the observed values (grey columns), the observed trend obtained from the Seasonal-Trend decomposition procedure (black line), and the regression line (red-slashed line) (the slashed grey line depicts the value of 600, which indicates risk of transmission). For an improved readability, it is suggested to visualize this figure in its digital version.

In Gobernador Gregores (located in the area where the recent fascioliasis spread has occurred, between latitudes 48° and 50° S), mean maximum and mean environmental temperatures increased significantly, mainly in summer (*p-value *< 0.001) (Fig 7a3 and 7b3). Regarding precipitation, it decreased (*p-value *< 0.001) (Fig 6e), especially during summer (*p-value *< 0.01) (Fig 7d3). Situated at higher elevation and further inland, Gobernador Gregores experiences a colder and more arid climate, which may historically have limited fascioliasis transmission. However, the longitudinal analysis of monthly Wb-bs values demonstrated a significant increment (*p-value* <0.001) (Figs 6i and 8 Panel A2), evidenced during every season excepting winter (*p-value *< 0.001) (Fig 7e1, 7e2, 7e3 and 7e4). Transmission risk values (>600) were sporadically first surpassed during the 1970s, increasing its magnitude and frequency since the 1990s (Fig 8 Panel A2), and specially evidenced during the last decade (Fig 8 Panel A3).

On the other hand, in San Julian (also located between latitudes 48° and 50° S but situated at lower elevation and closer to the Atlantic coast), the temperature increased significantly (as did the daily temperature range and the number of summer days) (Fig 6a, 6b, 6c, 6d and 6f), while precipitation remained stable (Fig 6e). The milder, slightly wetter coastal climate of San Julián provides more favorable conditions for intermediate snail host survival and habitat persistence compared to the interior. Furthermore, during the six-decade assessed period (1956–2021), 21 days were added to the transmission period (Fig 6i). Therefore, the Wb-bs values increased significantly (*p-value* <0.001) (Fig 6i), which is evidenced in every season except for the winter (*p-value* <0.001) (Fig 7e1, 7e2, 7e3 and 7e4). Transmission risk values, surpassing the threshold of 600, are consistently reached since the 1980s (Fig 8 Panels A2 and A3).

### 3.3. Analysis of the southern geographical spread of the disease

**Fig 9:**
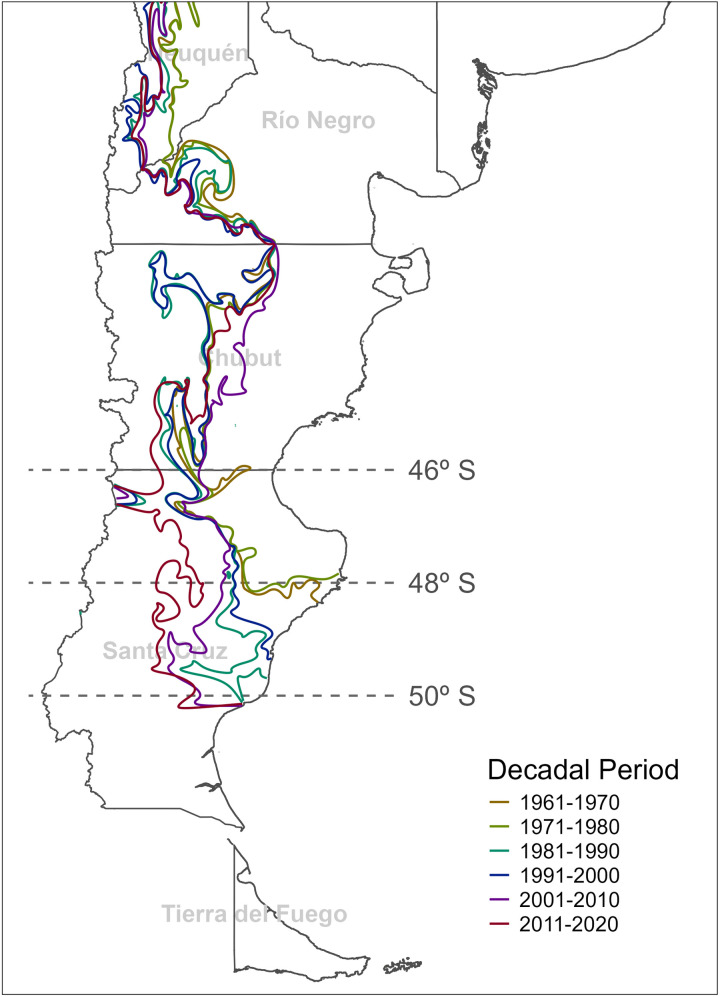
Southern spread during the 65-year period analyzed (1956-2021) of the fascioliasis endemic area as delimited by the Water-budget-based system (Wb-bs) climatic forecast index. Contour lines per decade represent the minimum Wb-bs value required for transmission to occur (Wb-bs transmission threshold = 600), obtained from interpolated continuous layers representing Wb-bs mean maximum values per decade. Country’s geopolitical shapes from GADM are freely available for academic use and other non-commercial use (https://gadm.org/license.html).

The analysis of the decadal geographical fascioliasis transmission threshold (Wb-bs index value = 600) by means of spatial interpolation techniques clarified the southern progression of the disease (Figs 9 and S1). As demonstrated in the longitudinal analyses of the Wb-bs index, transmission values (Wb-bs index value ≥ 600) were already reached up to latitude 48° S between 1960 and 1980 in the eastern part of the Patagonia region (Fig 9). From there, the spread of the suitable transmission area continues to the south and west of the Patagonia region, reaching the latitude 50° S by the beginning of the XIX century (Fig 9).

## 4. Discussion

This study demonstrates that climatic trends over the last six decades in Argentinean Patagonia, mainly characterized by rising temperatures, have increasingly favored conditions suitable for fascioliasis transmission in southern latitudes. By applying a modified Wb-bs index adapted for semi-arid environments, our analysis reveals that transmission risk values were reached with growing frequency since the 1990s, particularly in areas where recent outbreaks have occurred. This evidence suggests a strong link between climate variability and the emergence of fascioliasis in previously non-endemic areas.

The impact of global warming on the transmission of fascioliasis was highlighted during the 2010’s [17,18]. Since then, most of the studies dealing with the subject have focused on modeling future trends and potential shifts in geographic distribution (e.g., [60–65]). Yet, as far as we know, only a few have attempted to relate the observed climatic change with the uprise of outbreaks in endemic areas of animal [15] or human fascioliasis [7,14,66,67]. Indeed, assessing the impact of a changing climate on the spread of fascioliasis is extremely challenging, because it presents a worldwide distribution [13] with the widest latitudinal, longitudinal and altitudinal distribution known for a snail-borne disease [68]. For instance, a recent study demonstrated the climate-related geographical expansion of the human fascioliasis hyperendemic area in the Northern Bolivian Altiplano [16], whereas the unprecedented country-wide human fascioliasis outbreak in Vietnam could not be related to climate change [7]. Further, findings from other snail-borne diseases underscore the global relevance of this matter: for example, higher temperatures and increased rainfall were positively associated with the incidence of clonorchiasis in Guangzhou (China) [69]; and immediate and long-term rainfall, as well as medium-term temperature changes, influenced the emergence of neural angiostrongyliasis in dogs from 2020 to 2024 in eastern Australia [70].

Argentina has evidenced major climatic changes during the last 50 years. The situation baseline outlined in the 3^rd^ National Communication to the UNFCC (United Nations Framework Convention on Climate Change) [71], and later adopted in the country’s National Adaptation Plan [72], indicates that, throughout the period 1960–2010, in central and northern Argentina temperature has increased less than the mean global increase (< 0.5 °C). However, it also highlights that in Patagonia temperature has increased more than in the rest of the country, surpassing 1 °C in some places [71,73]. In fact, mean maximum temperatures mostly decrease over time in summer over northern Argentina, although they increase in Patagonia (southern Argentina) [74]. This may underlie opposed results which have also been reported, including a strong cooling trend in most of the country, presumably attributable to an increase of precipitation [75]. Regarding precipitation, it has certainly increased in most of the country, but with regional differences and strong interannual variations. Major increases have been evidenced in the east, while a decrease is observed along the central and southern Andean range [71,76]. In addition, extreme climatic events, such as heat or cold waves, heavy rains and floods, are increasingly frequent, with many historical records surpassed in recent years [76].

The analysis of seasonal-trend decomposition demonstrated a remarkable climatic change in the Patagonia region throughout the period assessed. The general and seasonal temperature increment detected fits well with previous studies which have already reported a pronounced warming process in the region [74,77,78]. Further, the increment in the frequency of summer days have also been reported before [79]. Indeed, the severity of regional heat waves has increased significantly during the last decades [80]. Concerning precipitation, the results obtained are consistent with previous reports. The positive precipitation trends detected along the Patagonian coast agree with the reported decrease in the occurrence of dry days (days without precipitation) in the area [81], while the precipitation decline in northern Patagonia near the Andes has been previously described [76,82].

These climatic shifts in Patagonia (particularly altered precipitation patterns, and rising temperatures) have significantly impacted livestock management and animal health, with broader socioeconomic repercussions. Increasing aridity, desertification, declining water availability, and changes in vegetation composition have led to reduced forage quality and quantity [83–85] with anticipated negative consequences on livestock productivity [86]. These ecological transformations have, in turn, necessitated adaptations in grazing practices, such as reductions in stocking density and the abandonment of historically productive areas [83,87]. Moreover, these climate-induced changes have contributed to the depopulation of rural areas, and increased economic vulnerability among Patagonian livestock producers [87].

The detailed analysis of the climatic forecast indices demonstrated a markedly seasonal pattern favoring the viability of fascioliasis transmission in the Patagonian region. When modified for its use in semi-desert and desert environments, the Wb-bs index reached values over 600 and demonstrated that fascioliasis transmission is possible from mid-summer (January-February) to late-autumn (May-June) in most of the region, as previously suggested based on empirical observations [34]. In the area where the recent fascioliasis spread has occurred, between latitudes 48° and 50° S, the Wb-bs index indicates that the threshold value (= 600) was occasionally reached before the 1980s but not allowing for a sustained transmission through time. Yet, during the last three decades, the Wb-bs index has shown a clear increasing trend, frequently reaching and surpassing the value of 600, thereby allowing disease transmission to occur almost every year between February and mid-March. This proposed threshold value of 600 serves as a widely recognized benchmark validated in several endemic regions across the Americas, Africa, Asia and Oceania (e.g., [14,16,42,48,50,51,64,66]), thus aligning the findings from the Patagonian region with epidemiological patterns observed globally.

In contrast, the Mt index, which is based on meteorological “wet days” (i.e., a day with precipitation > 1 mm) [88], proved to be unuseful to analyze the disease occurrence in the Patagonia region. The very low Mt values obtained indicated that the disease should not occur in the region, whereas it is a fact that transmission is occurring there. Originally devised for the Anglesey County [44] and extended for its use in the United Kingdom [89], the Mt or “Wet day” method relies only on precipitation to calculate a monthly value of wetness and forecast fascioliasis transmission [88]. Our results demonstrate that its use in semi-desert and desert environments, with limited rainfalls and a negative water balance (see [90]), is not advised, as in such conditions transmission may still proceed in permanent water collections [29]. Indeed, the permanently humid meadows and wetlands (*mallines*) constitute a highly valued grazing resource in local sheep production systems during spring and summer [86,91], which has in turn been associated with fascioliasis transmission [34]. Hence, a prior and cautious assessment of the local climatology and disease epidemiology is strongly recommended to avoid the inaccurate calculation of forecast indexes leading to potentially flawed interpretations.

The use of spatial interpolation techniques to determine the decadal geographical extent of the fascioliasis transmission area allowed to clarify the southern spread of the disease. Based on the values of the Wb-bs index, our analyses indicate that fascioliasis transmission was possible in north-eastern Santa Cruz province, between latitudes 46° and 48° S, already in the period 1960–1980. From there, in the following decades, the transmission area gradually expanded to the west and south, enabling the extension of the endemic area to the central plateau of Santa Cruz province and reaching the latitude 50° S. The field description of *F. hepatica* infected non-migrant sheep in the area [35] supports this modelling approach based on the Wb-bs index, as the geographical extent of the predicted transmission area accurately overlaps with the aforementioned empirical data.

Phenological models, such as the Wb-bs index used in this study, calculate growing degree-days above a threshold temperature to estimate a numerical value indicative of the disease transmission risk [92]. In this particular case the mean temperature threshold is assumed to be 10 °C, as it is the minimum temperature required for *F. hepatica* to develop in larval stages [49] and because lower temperatures inhibit the growth and reproduction of the transmitting lymnaeid snails [93]. However, the southern expansion of the fascioliasis endemic area in Patagonia is not completely explained by the significant increase of mean temperatures. Differing from the spread of the human fascioliasis endemic area in the Northern Bolivian Altiplano [94,95], where the increment of mean temperatures above 10 °C enabled lymnaeid snails to settle in higher grounds [16], in the Patagonia region mean temperatures normally exceed the proposed threshold during at least four months of the year (late-spring to early-autumn). Instead, such spread of the fascioliasis transmission area might be related with the regional increment in the frequency of days with mean temperature above 10 °C. For instance, in the locality of San Julian, the number of days with mean temperature exceeding 10 °C increased by 0.33 days per year (a total of 21 days in the 65-year period analyzed). This means that growing degree-days are accumulated during a larger period, and thus the risk of fascioliasis being transmitted increases accordingly.

It should be taken into account that some discrepancies may arise between the predicted geographical spread and the field observations:

- Inner zones of an endemic area may climatically evolve differently, as recently revealed for the Northern Bolivian Altiplano (see [96]). Despite the apparent physiographic homogeneity of the endemic flatland corridors in this human fascioliasis hyperendemic area, climate and its impact on fascioliasis transmission were proven to be markedly heterogeneous and locally influenced by physiographical features (e.g., altitude, closeness to inner hills and Lake Titicaca, and El Niño Southern Oscillation) [96].
- A time-lag may appear between the presence of suitable climatic conditions for fascioliasis to occur in Argentina between latitudes 46° and 48° S, already in 1960’s, and the first description of parasitized animals in the area (reported by Olaechea, 2007 [32]). This time-lag might be attributed to: (i) the time needed for fasciolids and lymnaeids to disperse, reach and establish in a new area; for instance, in the Northern Bolivian Altiplano, a much smaller geographical area, new lymnaeid populations were described in higher altitude places almost 30 years after temperature was high enough to allow their long-term survival [16,95]; (ii) the ability to detect an unexpected disease in a non-endemic area; for instance, for the inexpert eye, some of the lesions and symptomatology caused by liver fluke infection could be easily mistaken with those of a heavy infection by *Thysanosoma actinoides*, a tapeworm very frequently reported in cattle and sheep in the Patagonia region [35,97].

## 5. Concluding remarks

This study presents the first comprehensive analysis of the southward expansion of fascioliasis in South American Patagonia, demonstrating a clear link between this spread and recent climatic shifts. Rising temperatures and an extended duration of biologically permissive conditions have led to increasingly favorable environmental conditions for the parasite’s life cycle and the persistence of intermediate snail hosts, particularly in areas between 48° and 50° S. The study also provides some crucial recommendations and concerns regarding the application and interpretation of two widely used climatic forecast indices (Mt and Wb-bs) in arid and semi-arid regions. If current climate trends persist, this geographical expansion is expected to progress further. The identification of a seasonal transmission window highlights a key timeframe for implementing targeted control measures (e.g., timely anthelmintic treatments, enhanced surveillance, and intensified diagnostic monitoring) to mitigate infection risk and alleviate the economic impact on livestock producers. These findings not only provide critical insight into local disease dynamics but also underscore the broader implications of climate-driven changes in the distribution of snail-borne diseases globally.

## Supporting information

S1 Table Mean monthly values ± standard deviation, and (ranges) for climatic factors recorded at selected meteorological stations in the area of interest. (DOCX)

S1 Fig Decadal fascioliasis transmission area obtained by spatial interpolation techniques. (JPEG)
