# Supplementary material for: Impact of climate change on the spread of fascioliasis into the extreme south of South America
Source: PLoS Negl Trop Dis. 2025 Aug 18;19(8):e0013433. doi: 10.1371/journal.pntd.0013433 (PMC12377589; doi:10.1371/journal.pntd.0013433)
Supplement: S1 Table — (DOCX) [file pntd.0013433.s001.docx]

Running Title: Climate change impact on the spread of a snail-borne disease to extreme latitudes

Running authors: P.F. Cuervo *et al.*

_________________________________________

**Impact of climate change on the snail-borne zoonotic parasitic disease of fascioliasis spreading into areas of extreme latitude**

**Pablo F. Cuervo ^1,2,^*, Roberto Mera y Sierra ^3^, Patricio Artigas ^1,2^, M. Cecilia Fantozzi ^1,2^, María Dolores Bargues ^1,2^, Santiago Mas-Coma ^1,2^**

**^1^** Departamento de Parasitología, Facultad de Farmacia, Universidad de Valencia, Av. Vicent Andrés Estellés s/n, Burjassot, Valencia, Spain.

**^2^** CIBER de Enfermedades Infecciosas, Instituto de Salud Carlos IIII, C/ Monforte de Lemos 3-5. Pabellón 11. Planta 0, Madrid, Spain.

^3^ Centro de Investigación en Parasitología Regional (CIPAR), Universidad Juan Agustín Maza, Avenida Acceso Este, Lateral Sur 2245, Guaymallén, Mendoza, Argentina.

*** Corresponding author:**

Dr. Pablo F. Cuervo

Email: Pablo.F.Cuervo@uv.es

**Table S1.** Mean monthly values ± standard deviation, and (ranges) for climatic factors recorded at selected meteorological stations in the area of interest.

| **Variable** | **Neuquén**  **(1956-2021)** | **Río Colorado**  **(1956-2019)** | **San Antonio Oeste**  **(1988-2019)** | **Viedma**  **(1967-2021)** | **Puerto Madryn**  **(1992-2019)** | **Maquinchao**  **(1956-2021)** |
| --- | --- | --- | --- | --- | --- | --- |
| **MET (°C)** | 15.1±6 (3.3-26.1) | 16±5.6 (5.4-27) | 15±5.6 (4.6-25.2) | 14.5±5.3 (4.9-24.6) | 13.5±5.1 (3.6-22.4) | 9.6±5.8 (-4.2-20.8) |
| **MMT (°C)** | 22.5±6.7 (8.5-33.8) | 22.9±6.4 (9.6-35.4) | 21.9±6.1 (10.3-32.7) | 21.2±5.9 (9.7-32.3) | 20±5.8 (8.6-30.5) | 16.8±7 (0.6-29.2) |
| **MmT (°C)** | 7.6±5.4 (-3.8-18.4) | 9.1±4.9 (-1.4-18.9) | 8.1±5.2 (-1.3-18.5) | 7.8±4.7 (-1.1-17.6) | 7±4.5 (-2.1-15.8) | 2.4±4.8 (-8.9-12.5) |
| **EMT (°C)** | 30.1±6.4 (15.1-42.3) | 30.5±6.6 (15.4-42.8) | 30.7±7.1 (15.6-43.5) | 29.6±7.2 (14-43.7) | 28.6±6.4 (15.4-43.4) | 24.8±7.4 (5.6-38.9) |
| **EmT (°C)** | 7.6±5.4 (-3.8-18.4) | 9.1±4.9 (-1.4-18.9) | 8.1±5.2 (-1.3-18.5) | 7.8±4.7 (-1.1-17.6) | 7±4.5 (-2.1-15.8) | 2.4±4.8 (-8.9-12.5) |
| **MTD (°C)** | 14.8±2 (5.8-19.8) | 13.7±2.2 (6.8-18.4) | 13.7±1.8 (8-18.5) | 13.4±1.9 (7.9-18.3) | 12.9±1.8 (7.9-17.2) | 14.3±2.9 (6.3-20.8) |
| **ETD (°C)** | 16.8±2.8 (9.2-26.6) | 15.8±2.6 (8.5-24.9) | 18.3±3.1 (9.9-26.3) | 17.4±2.7 (10.3-25.3) | 15.8±2.9 (9-27.8) | 17.4±3 (10.5-27.8) |
| **Pt (mm)** | 16.7±23.4 (0-228.7) | 39.1±38.3 (0-270.4) | 24±27.3 (0-210) | 31.3±30.7 (0-215.9) | 17.6±24.6 (0-220.5) | 16.5±19.8 (0-178.5) |
| **YP (mm)** | 199.6±92.1 (60.4-476.7) | 418.9±157.4 (87.1-784.9) | 282.9±120 (73.9-577.8) | 362.2±126 (25.5-652.8) | 206.6±84.4 (63.3-353.6) | 196.5±73.5 (54.1-393.3) |
| **MP (mm)** | 65.2±41.6 (0-228.7) | 114.2±46.7 (35.5-270.4) | 77.7±40.4 (21.8-210) | 93.3±42.7 (14.9-215.9) | 71.3±45.4 (20.5-220.5) | 56.9±29.6 (14.9-178.5) |
| **DP (days)** | 2±2.2 (0-10) | 4±2.4 (0-12) | 3±2.1 (0-11) | 4±2.4 (0-15) | 2±2.1 (0-10) | 2±2.3 (0-12) |
| **DF (days)** | 0±0.2 (0-1) | 0±0.1 (0-1) | 0±0.1 (0-1) | 0±0.1 (0-1) | 0±0.2 (0-1) | 0±0.5 (0-1) |
| **PET (mm)** | 145.3±84.5 (25.7-409.7) | 125.7±78.3 (24.5-350.8) | 137.3±87.4 (25.8-363) | 127.5±79.7 (25-321.9) | 101.8±67 (17.9-264.8) | 125.9±88.2 (13.9-362.5) |
| **Mt** | 2.1±7.3 (0-91.1) | 7.7±13.3 (0-112) | 3.5±9.6 (0-119.5) | 5±10.7 (0-72) | 2.8±7.8 (0-63.6) | 0.6±2.7 (0-36) |
| **Wb-bs** | 796.1±810.7 (0-2500.8) | 793.7±821.5 (0-3411.7) | 743.1±763.5 (0-2346.9) | 706.9±723.7 (0-2320) | 491.6±573.2 (0-1704.2) | 230.9±329.7 (0-1209.3) |
| **Variable** | **Bariloche**  **(1956-2021)** | **El Bolsón**  **(1978-2019)** | **Esquel**  **(1961-2021)** | **Trelew**  **(1956-2021)** | **Paso de Indios**  **(1968-2019)** | **Comodoro Rivadavia**  **(1956-2021)** |
| **MET (°C)** | 8.4±4.4 (-1.5-18.3) | 10.4±4.5 (2.1-19.5) | 8.5±4.7 (-2.9-18.4) | 13.9±5.3 (3.5-23.5) | 10.6±5.3 (-2.2-21.4) | 13.2±4.6 (3.2-21.6) |
| **MMT (°C)** | 14.5±5.9 (2.7-27.4) | 17.1±6.1 (5.8-30) | 14.4±5.6 (1.2-26.4) | 20.7±6 (8.1-31.5) | 17.7±6.4 (2.8-29.9) | 18.5±5.5 (7-28.8) |
| **MmT (°C)** | 2.3±3.1 (-5.8-10) | 3.6±3.1 (-3.6-10.4) | 2.6±3.9 (-7.1-11.6) | 7.2±4.7 (-2.7-15.9) | 3.6±4.5 (-7.3-13.3) | 8±3.8 (-0.6-15.2) |
| **EMT (°C)** | 22.1±7 (7.5-35.4) | 24.1±6.9 (10.7-37.5) | 22±6.4 (7.3-35.6) | 29.4±6.8 (14-42.2) | 25.7±7.1 (8.1-39.9) | 26.6±6.5 (12.1-40.2) |
| **EmT (°C)** | 2.3±3.1 (-5.8-10) | 3.6±3.1 (-3.6-10.4) | 2.6±3.9 (-7.1-11.6) | 7.2±4.7 (-2.7-15.9) | 3.6±4.5 (-7.3-13.3) | 8±3.8 (-0.6-15.2) |
| **MTD (°C)** | 12.3±3.4 (5.2-20.4) | 13.6±3.5 (5.9-20.9) | 11.8±2.2 (6.7-17.4) | 13.6±1.9 (7.9-18.6) | 14.1±3 (7.3-23.2) | 10.5±1.9 (5.7-14.8) |
| **ETD (°C)** | 18.6±3.5 (7.2-26.6) | 16±3.3 (8.1-25) | 15.8±2.8 (8.4-24.7) | 16.6±2.6 (8.6-24.5) | 15.9±3.2 (8.4-25.4) | 13.3±3.1 (6-24.3) |
| **Pt (mm)** | 66.5±68.7 (0-399.7) | 76.8±73.5 (0-397) | 40.9±38.3 (0-223.5) | 16.7±20.5 (0-244.8) | 15.5±18.9 (0-134.9) | 20.5±26.8 (0-320.4) |
| **YP (mm)** | 784.7±197.6 (183.1-1208.6) | 788.9±305.2 (52.8-1338.8) | 480.5±119.6 (174.8-707.4) | 200.4±71.6 (78.8-368.6) | 168.2±74.9 (30.9-376.2) | 243.1±94.2 (90.2-561.2) |
| **MP (mm)** | 209±72.3 (0-399.7) | 221.8±81.2 (52.8-397) | 122.6±39.4 (49.8-223.5) | 58.8±37.7 (20.1-244.8) | 53.8±28.3 (14.3-134.9) | 78.7±46.3 (22.1-320.4) |
| **DP (days)** | 6±5 (0-22) | 7±5 (0-26) | 5±3.6 (0-20) | 2±2.2 (0-12) | 2±2.5 (0-13) | 2±2.3 (0-14) |
| **DF (days)** | 0±0.4 (0-1) | 0±0.3 (0-1) | 0±0.5 (0-1) | 0±0.2 (0-1) | 0±0.4 (0-1) | 0±0.1 (0-1) |
| **PET (mm)** | 119.3±87.5 (17.6-427.2) | 123.8±98.6 (16.9-441.3) | 92.8±60.5 (13.9-256.3) | 122.3±79.5 (20-342.4) | 118.5±89.9 (13.5-495.5) | 86.6±54.6 (17.2-239.7) |
| **Mt** | 0.9±5.5 (0-94.8) | 3.7±13 (0-140.2) | 1.3±4.7 (0-52.8) | 2.2±6.2 (0-69.8) | 1±4.6 (0-55.1) | 3.8±9.5 (0-129.7) |
| **Wb-bs** | 98.9±166 (0-668.9) | 212.5±296.9 (0-1023.4) | 111.1±179.2 (0-754.2) | 630.5±662.3 (0-1915.7) | 278.9±379.8 (0-1234.6) | 518.4±558.5 (0-2043.3) |
| MET, mean environmental temperature; MMT, mean maximum temperature; MmT, mean minimum temperature; EMT, extreme maximum temperature, EmT, extreme minimum temperature; MTD, maximum temperature difference; ETD, extreme temperature difference; Pt, precipitation; YP, yearly precipitation; MP, maximum precipitation; DP, number of days with precipitation; DF, number of days with freeze; PET, potential evapotranspiration; Mt, wet-day index; Wb-bs, water-based-budget index. | | | | | | |
|  | | | | | | |
| (cont…) | | | | | | |

| (cont…) |  |  |  |  |  |  |  |
| --- | --- | --- | --- | --- | --- | --- | --- |
|  |  |  |  |  |  |  |  |
| **Variable** | **Perito Moreno**  **(1956-2018)** | **Puerto Deseado**  **(1956-2019)** | **Gobernador Gregores**  **(1956-2019)** | **San Julián**  **(1956-2021)** | **Santa Cruz**  **(1958-2019)** | **El Calafate**  **(2000-2019)** |  |
| **MET (°C)** | 8.8±4.8 (-3.4-18.5) | 10.2±4.6 (0-18.7) | 8.8±5.1 (-6.6-19) | 9.9±4.7 (-0.6-18.7) | 8.8±4.9 (-2.9-17.3) | 7.1±4.6 (-4.2-15.4) |  |
| **MMT (°C)** | 14±5.4 (1.2-25.8) | 15.4±5.6 (4.2-25.6) | 14.4±6.1 (-1.7-26.4) | 15.3±5.7 (2.6-26.3) | 14.3±6 (0.9-24.9) | 12.8±5.1 (0.4-22.1) |  |
| **MmT (°C)** | 3.4±4.4 (-10.2-11.9) | 5.2±3.7 (-4.1-12.7) | 3.2±4.3 (-11.5-11.7) | 4.5±3.7 (-3.8-11.6) | 3.3±3.8 (-6.9-10.3) | 1.5±4.2 (-8.8-9.1) |  |
| **EMT (°C)** | 20.6±6.1 (7.6-35) | 23.6±7.1 (7.9-40.1) | 21.9±6.8 (6.3-38.5) | 23.7±7.1 (6.9-37.5) | 21.8±7.1 (4.8-37) | 19±5 (6.6-28.4) |  |
| **EmT (°C)** | 3.4±4.4 (-10.2-11.9) | 5.2±3.7 (-4.1-12.7) | 3.2±4.3 (-11.5-11.7) | 4.5±3.7 (-3.8-11.6) | 3.3±3.8 (-6.9-10.3) | 1.5±4.2 (-8.8-9.1) |  |
| **MTD (°C)** | 10.6±1.6 (6.3-16.1) | 10.2±2.2 (4.3-15.4) | 11.1±2.2 (5.9-16.8) | 10.9±2.3 (4.9-15.9) | 10.9±2.6 (4.8-15.8) | 11.3±1.5 (6.8-14.9) |  |
| **ETD (°C)** | 13.5±2.5 (7-21.7) | 12.9±3.3 (6-24.3) | 13±2.5 (5.3-21.4) | 13.5±3.4 (5-24.3) | 12.9±3.3 (5.5-20.9) | 12.9±2 (8.1-20.2) |  |
| **Pt (mm)** | 12.6±13.6 (0-88.5) | 17.6±16.7 (0-125.9) | 12.2±12.2 (0-85.3) | 21.3±21 (0-181.9) | 16.9±16 (0-128.2) | 13.6±14.7 (0-107.1) |  |
| **YP (mm)** | 121.8±61 (1-256.1) | 206.1±67.6 (96.3-385.7) | 129.2±61.3 (16.8-290.8) | 236.8±100.7 (46.3-503) | 154.1±76.9 (6.2-367.4) | 154.9±65.9 (11-272.9) |  |
| **MP (mm)** | 36.4±19 (1-88.5) | 50.9±23.6 (19.6-125.9) | 35.2±17.2 (8.5-85.3) | 62.2±30.1 (18.3-181.9) | 42.3±23.9 (0-128.2) | 40.9±21.7 (11-107.1) |  |
| **DP (days)** | 2±2.3 (0-10) | 3±2.6 (0-14) | 2±2.1 (0-12) | 3±2.5 (0-13) | 3±2.5 (0-13) | 2±2.1 (0-10) |  |
| **DF (days)** | 0±0.4 (0-1) | 0±0.2 (0-1) | 0±0.4 (0-1) | 0±0.3 (0-1) | 0±0.3 (0-1) | 0±0.5 (0-1) |  |
| **PET (mm)** | 68.6±43.9 (9.9-198.5) | 70.5±48.4 (10.8-179.7) | 72.7±51.8 (7.5-266.4) | 78.1±55.3 (11.1-219.1) | 76.5±55.4 (9.2-211.6) | 66.2±43.9 (9.8-155) |  |
| **Mt** | 1±3.3 (0-47.4) | 3.1±7.1 (0-64.8) | 1.2±3.4 (0-36.9) | 2.9±7.1 (0-53.6) | 1.8±4.9 (0-28.6) | 1±3 (0-21.7) |  |
| **Wb-bs** | 119.4±190.4 (0-719) | 221.8±301.7 (0-1181.8) | 132.6±211.6 (0-821.1) | 204.1±274.9 (0-945.8) | 125.4±193.1 (0-712.1) | 59.1±103.5 (0-450.9) |  |
| **Variable** | **Río Gallegos**  **(1956-2021)** | **Río Grande**  **(1959-2021)** | **Ushuaia**  **(1990-2021)** |  |  |  |  |
| **MET (°C)** | 8±4.5 (-2.8-16) | 5.8±3.9 (-6.2-13.3) | 6.2±3 (-2.1-12.4) |  |  |  |  |
| **MMT (°C)** | 13.1±5.5 (0.9-23) | 10.1±4.7 (-1.7-18.2) | 9.6±3.6 (2.1-17) |  |  |  |  |
| **MmT (°C)** | 2.8±3.5 (-6.4-9.8) | 1.4±3.2 (-10.6-8.3) | 2.7±2.6 (-6.4-8.4) |  |  |  |  |
| **EMT (°C)** | 20±6.7 (5.2-35.8) | 15.5±5.7 (2.3-30.8) | 16.3±4.8 (5.6-28.5) |  |  |  |  |
| **EmT (°C)** | 2.8±3.5 (-6.4-9.8) | 1.4±3.2 (-10.6-8.3) | 2.7±2.6 (-6.4-8.4) |  |  |  |  |
| **MTD (°C)** | 10.3±2.3 (4.5-15.3) | 8.8±1.9 (4.6-13.5) | 6.9±1.9 (3.1-11.7) |  |  |  |  |
| **ETD (°C)** | 12.3±3 (5.1-24.2) | 11±2.4 (5.6-19.4) | 9.3±2.4 (2.8-18.3) |  |  |  |  |
| **Pt (mm)** | 21±16.6 (0-98) | 27.5±19.9 (0-132) | 41.5±24.4 (0.8-178.2) |  |  |  |  |
| **YP (mm)** | 242.8±76 (37.6-419.6) | 300.4±120.4 (4-537.9) | 464.4±148.3 (138.6-820.6) |  |  |  |  |
| **MP (mm)** | 52.7±18.3 (0-98) | 61.9±26 (4-132) | 85.6±30.5 (44.1-178.2) |  |  |  |  |
| **DP (days)** | 4±2.9 (0-17) | 6±3.2 (0-17) | 9±3.4 (0-19) |  |  |  |  |
| **DF (days)** | 0±0.4 (0-1) | 0±0.5 (0-1) | 0±0.3 (0-1) |  |  |  |  |
| **PET (mm)** | 66.6±46.3 (8.4-176.5) | 52.9±36.5 (5.7-130.9) | 51±34.6 (6.2-122.9) |  |  |  |  |
| **Mt** | 3.9±8.6 (0-59.1) | 2.9±8.8 (0-73.9) | 1.7±6.4 (0-46.2) |  |  |  |  |
| **Wb-bs** | 85±134.4 (0-530.3) | 7.6±21.6 (0-160.2) | 2.5±10.1 (0-105.4) |  |  |  |  |
| MET, mean environmental temperature; MMT, mean maximum temperature; MmT, mean minimum temperature; EMT, extreme maximum temperature, EmT, extreme minimum temperature; MTD, maximum temperature difference; ETD, extreme temperature difference; Pt, precipitation; YP, yearly precipitation; MP, maximum precipitation; DP, number of days with precipitation; DF, number of days with freeze; PET, potential evapotranspiration; Mt, wet-day index; Wb-bs, water-based-budget index. | | | | | | | |
