# Supplementary figures and images for: Impact of climate change on the spread of fascioliasis into the extreme south of South America
Source: PLoS Negl Trop Dis. 2025 Aug 18;19(8):e0013433. doi: 10.1371/journal.pntd.0013433 (PMC12377589; doi:10.1371/journal.pntd.0013433)

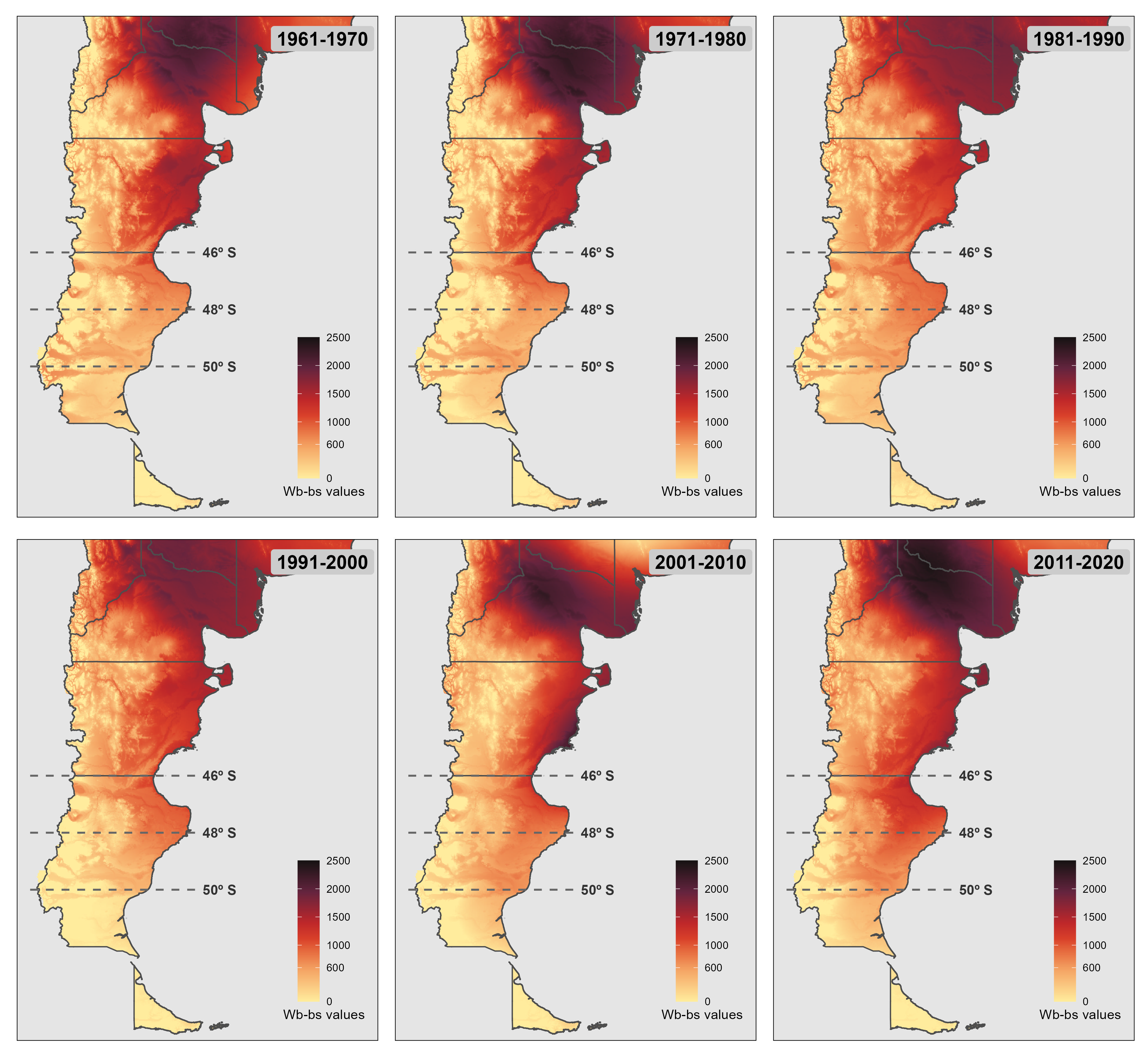

Supplement: S1 Fig — (JPEG) [file pntd.0013433.s002.jpeg]
